# Freezing Tolerance of *Lolium multiflorum/Festuca arundinacea* Introgression Forms is Associated with the High Activity of Antioxidant System and Adjustment of Photosynthetic Activity under Cold Acclimation

**DOI:** 10.3390/ijms21165899

**Published:** 2020-08-17

**Authors:** Adam Augustyniak, Izabela Pawłowicz, Katarzyna Lechowicz, Karolina Izbiańska-Jankowska, Magdalena Arasimowicz-Jelonek, Marcin Rapacz, Dawid Perlikowski, Arkadiusz Kosmala

**Affiliations:** 1 Institute of Plant Genetics, Polish Academy of Sciences, Strzeszyńska 34, 60-479 Poznań, Poland; aaug@igr.poznan.pl (A.A.); ipaw@igr.poznan.pl (I.P.); kmas@igr.poznan.pl (K.L.); dper@igr.poznan.pl (D.P.); 2 Department of Plant Ecophysiology, Institute of Experimental Biology, Faculty of Biology, Adam Mickiewicz University, Uniwersytetu Poznańskiego 6, 61-614 Poznań, Poland; karolina.izbianska@amu.edu.pl (K.I.-J.); arasim@amu.edu.pl (M.A.-J.); 3 Department of Plant Breeding, Physiology and Seed Science, University of Agriculture in Kraków, Podłużna 3, 30-239 Kraków, Poland; rrrapacz@cyf-kr.edu.pl

**Keywords:** antioxidant system, cold acclimation, *Lolium-Festuca*, photosynthesis, freezing tolerance, forage grasses

## Abstract

Though winter-hardiness is a complex trait, freezing tolerance was proved to be its main component. Species from temperate regions acquire tolerance to freezing in a process of cold acclimation, which is associated with the exposure of plants to low but non-freezing temperatures. However, mechanisms of cold acclimation in *Lolium-Festuca* grasses, important for forage production in Europe, have not been fully recognized. Thus, two *L. multiflorum/F. arundinacea* introgression forms with distinct freezing tolerance were used herein as models in the comprehensive research to dissect these mechanisms in that group of plants. The work was focused on: (i) analysis of cellular membranes’ integrity; (ii) analysis of plant photosynthetic capacity (chlorophyll fluorescence; gas exchange; gene expression, protein accumulation, and activity of selected enzymes of the Calvin cycle); (iii) analysis of plant antioxidant capacity (reactive oxygen species generation; gene expression, protein accumulation, and activity of selected enzymes); and (iv) analysis of Cor14b accumulation, under cold acclimation. The more freezing tolerant introgression form revealed a higher integrity of membranes, an ability to cold acclimate its photosynthetic apparatus and higher water use efficiency after three weeks of cold acclimation, as well as a higher capacity of the antioxidant system and a lower content of reactive oxygen species in low temperature.

## 1. Introduction

Environmental stresses, both abiotic and biotic, are the principal factors affecting plant growth and development. Furthermore, plants, including crops, are often exposed simultaneously to multiple stresses, reducing significantly their yield potential [1]. Winter stresses are among the major environmental conditions, which affect plants. Winter-hardiness, a trait which enables plants to survive the winter period, is associated with a plant’s capacity to overcome a wide spectrum of unfavorable factors, such as frost, rapidly changing temperatures, light stress, associated with photoinhibition, wind, desiccation, anoxia, ice encasement, mechanical damage (soil freeze/de-freeze cycles), de-acclimation, or various winter-related diseases (e.g., snow molds). On the other hand, freezing tolerance is often thought to be the most crucial trait to determine levels of winter-hardiness, as it usually has the most significant impact on plant survival in winter [2].

Plants from cold and temperate regions, including grasses, are periodically exposed to sub-zero temperatures. These environmental conditions initiate acquiring freezing tolerance in the process of cold acclimation (CA). This phenomenon alters the expression of wide range of genes and is associated with many physiological and biochemical changes within plant cells, including modifications in structure and compositions of cellular membranes, spectrum of iso-enzymes and their activities, accumulation of numerous proteins, and contents of crucial metabolites [3,4]. It has been proved that a crucial role in CA both in perennial and annual plant species play the C-repeat binding factors (CBF), which are the transcription factors. They bind to the C-repeat sequence motifs in the promotor region of regulated genes [5]. Genes affected by CBF transcription factors are referred to as the CBF regulon [6]. The most crucial of these genes are the members of cold regulated (*Cor*) gene family [7]. Among them, *Cor14b* is the most recognized *Cor* gene in cereals [8,9,10]. The freeze-induced alterations in cellular membranes, which are the primary sites of cellular damage, result mainly from severe dehydration associated with water freezing in apoplast. However, membranes’ damage could be also a result of accumulation of reactive oxygen species (ROS). Although, these particles are important signaling compounds, in higher amounts, they are also damaging agents causing protein oxidation and lipid peroxidation. The homeostasis of ROS in plant cells depends on the control over ROS production, which is tightly associated with plant photosynthetic capacity, and ROS scavenging, associated with plant antioxidant efficiency [11,12]. A generation of hydrogen peroxide (H_2_O_2_), intensified during environmental stresses, was proved to be involved in the regulation of stomatal aperture [13,14,15,16], stress signalling and acclimation, regulation of gene expression [17], and programmed cell death [18]. Although superoxide anion radical (O_2_^•−^) is considered as an important messenger molecule in stress acclimation processes, it also acts as a trigger for other ROS generation, specifically H_2_O_2_ [17,19]. Chloroplasts and peroxisomes are the principal ROS producing organelles in plant cells. However, also the other compartments, including mitochondria, cytoplasm and apoplast, are involved in this phenomenon [19]. The balance between peroxidases, superoxide dismutases (SOD), and catalases (CAT) activities was proved to be essential for maintenance of redox homeostasis. Overproduced H_2_O_2_ is commonly catabolised by CAT and peroxidases. Catalases, present mainly in peroxisomes and glyoxysomes, are responsible for H_2_O_2_ decomposition to O_2_ and H_2_O. These two compartments are the primary sites of H_2_O_2_ generation and utilisation, but CAT can also be found in chloroplasts, mitochondria and cytosol [20]. Ascorbate peroxidase (APX) is the most important peroxidase in H_2_O_2_ detoxification, which uses two molecules of ascorbate to reduce H_2_O_2_ to water [21,22]. Glutathione peroxidases (GPX) are involved in H_2_O_2_ decomposition, using endogenous reduced glutathione (GSH) as a reducing factor. Guaiacol peroxidase (POD) oxidizes phenols at the expense of H_2_O_2_ and is located in cytosol, vacuole, cell wall, and apoplast, thus it is engaged in a wide range of processes related to ROS over-accumulation under stress conditions [23]. Superoxide dismutases constitute the frontline for O_2_^•−^ scavenging in all the compartments, where superoxide radicals are generated [24]. These enzymes act via dismutating O_2_^•−^ into O_2_ and H_2_O_2_. Plant SOD is found in mitochondria (Mn-SOD), chloroplasts (Fe-SOD and Cu/Zn-SOD), peroxisomes (Cu/Zn-SOD), and cytosol (Cu/Zn-SOD) [25]. It was demonstrated that CAT, peroxidases, and SOD revealed elevated accumulations and activities in response to unfavorable environmental conditions [26,27,28,29,30].

The *Lolium–Festuca* complex involves numerous species and hybrids, which have already been proved to be crucial for grassland production in Europe. Some of them have been also successfully used as excellent plant models to decipher mechanisms of tolerance to a wide range of environmental stress conditions, including winter stresses [31,32,33]. As demonstrated earlier, *F. arundinacea* (tall fescue) was shown to be a valuable source of genes governing abiotic and biotic stress tolerance to be transferred to closely related, high forage quality *L. multiflorum* (Italian ryegrass), with a rather limited capacity to withstand these environmental stress conditions. Applications of the research addressing *L. multiflorum*/*F. arundinacea* introgression forms, with the selected components of stress tolerance transferred from *F. arundinacea* into *L. multiflorum* genomic background (drought tolerance [34,35,36,37], and freezing tolerance [32,38,39]) allowed us to dissect these complex traits into their different components present in different introgression lines, and to further analyze these components separately and in detail.

Augustyniak et al. (2018) [38] suggested the steering role of photosynthetic performance in the process of CA in *L. multiflorum*/*F. arundinacea* introgression forms. The photochemical mechanism of photosynthetic adjustment, associated with the acclimation of photosynthetic apparatus to low temperature conditions in the high freezing tolerant (HFT) introgression form, was shown to be crucial to prevent the possible photoinhibition of photosynthesis under cold conditions. Furthermore, the levels of CO_2_ assimilation and metabolism in more freezing tolerant form, were suggested to be regulated in non-stomatal way and driven by the Calvin cycle efficiency, associated with the activity of chloroplastic aldolase. It was also assumed by Augustyniak et al. (2018) [38], based on a comprehensive proteomic study, that the HFT *L. multiflorum*/*F. arundinacea* introgression form could have a higher capacity of its cellular antioxidant system, compared to the low freezing tolerant (LFT) form, as the abundance of some cellular proteins, potentially involved in redox homeostasis/antioxidant activity, was higher under CA in the form with higher tolerance. These hypotheses were the starting points for our further study presented here to go deeper into the understanding of CA process in forage grasses.

Herein, we hypothesize, first of all, that the balance between photosynthetic performance and cellular antioxidant capacity under CA in forage grasses could be one of the most crucial components of freezing tolerance, manifested by the increase of cellular membranes’ integrity, in this group of plants. Furthermore, we also hypothesize that introgression forms with higher freezing tolerance could be characterized during CA by a lower content of ROS, simultaneously accompanied by higher accumulation and activities of antioxidant enzymes, and higher accumulation of Cor14b protein, which was postulated earlier not only to enhance combined freezing tolerance and resistance to photoinhibition but also to have ‘membrane-protective’ functions [9], as described earlier for its analog (Cor15a) in Arabidopsis [40,41].

Thus, our comprehensive study deciphered the relationships between (i) generation of ROS (O_2_^•-^ and H_2_O_2_), (ii) capacity of enzymatic antioxidant system (gene expression, protein accumulation, and activity of antioxidant enzymes: glutathione reductase (GR), GPX, APX, CAT, Fe-SOD, Cu/Zn-SOD, and Mn-SOD; and activity of POD), (iii) cellular membranes’ integrity (temperature causing a 50% electrolyte leakage (T_EL50_) and lipid peroxidation), and (iv) capacity of photosynthesis (chlorophyll fluorescence; gas exchange: stomatal conductance (*gs*), transpiration (*E*), and CO_2_ assimilation (*A*); gene expression and protein accumulation of two Calvin cycle enzymes: plastid fructose-1,6-bisphosphate aldolase (pFBA), crucial for the regeneration step of the cycle, and plastid phosphoglycerate kinase (pPGK), crucial for the reduction step of the cycle; as well as the activity of pPGK). Additionally, we analyzed (v) the accumulation of Cor14b protein. The research was performed at several time-points of CA conditions in two selected earlier introgression forms of *L. multiflorum*/*F. arundinacea*, distinct in their levels of freezing tolerance.

## 2. Results

### 2.1. T_EL50_ and Lipid Peroxidation

**Figure 1:**
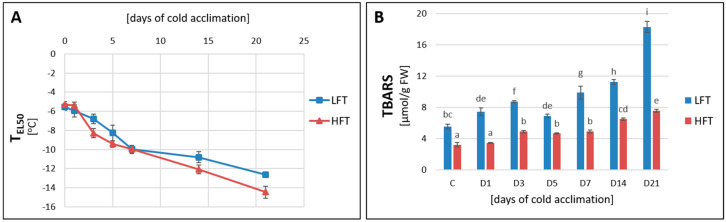
Changes in T_EL50_ (temperature causing a 50% electrolyte leakage-EL) (**A**) and thiobarbituric acid reactive substances (TBARS) content (**B**), at seven time-points: before cold acclimation (CA) C and on the 1st, 3rd, 5th, 7th, 14th, and 21st day of CA in *Lolium multiflorum/Festuca arundinacea* introgression forms. Error bars represent confidence intervals for *p* = 0.05 (**A**) and standard errors of five replicates (**B**). Homogeneity groups are denoted by the same letters, according to Fisher’s LSD test (*p* = 0.05). HFT–high freezing tolerant introgression form, LFT–low freezing tolerant introgression form; FW–fresh weight.

The estimation of T_EL50_ confirmed that CA process significantly and progressively increased stability of cellular membranes under freezing conditions in both analyzed introgression forms. However, after full time of acclimation, the HFT form revealed a lower value of T_EL50_ parameter (−14.47 °C), compared with the LFT form (−12.62 °C) (Figure 1A). Although, the level of thiobarbituric acid reactive substances (TBARS) raised significantly during CA also in both analyzed forms, it was significantly higher at each analyzed experimental time-point in the LFT form (Figure 1B).

### 2.2. Chlorophyll Fluorescence

**Figure 2:**
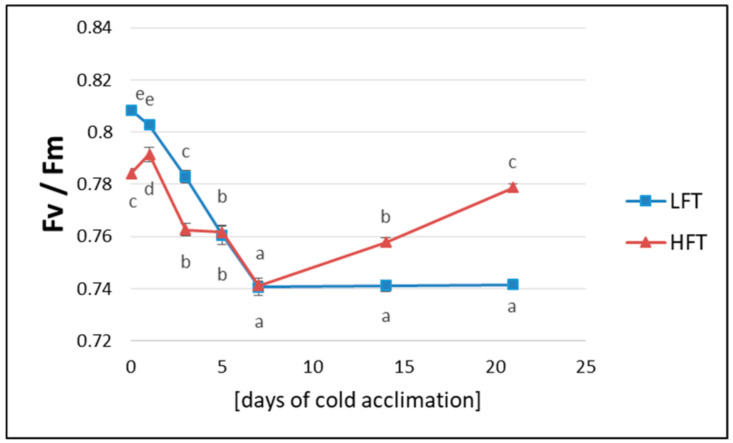
Changes in maximum quantum yield of primary PSII photochemistry (*Fv*/*Fm*) (yield ratio was calculated for *t* = 0) in the high (HFT) and the low freezing tolerant (LFT) *Lolium multiflorum/Festuca arundinacea* introgression forms, at seven time-points: before cold acclimation (CA) and on the 1st, 3rd, 5th, 7th, 14th, and 21st day of CA. Error bars represent the standard errors of ten replicates. Homogeneity groups are denoted by the same letters, according to Fisher’s LSD test (*p* = 0.05).

As shown in Figure 2, the parameter of maximum quantum yield of primary photosystem II (PSII) photochemistry (*Fv/Fm*), in the case of the LFT form, decreased significantly and progressively during seven days of CA and remained at this lower level until the end of the CA process. On the other hand, the HFT form revealed only a temporal decrease of this parameter and starting from the 7th day of CA, *Fv/Fm* increased significantly, achieving a higher value after full time of CA (Figure 2).

### 2.3. Gas Exchange

**Figure 3:**
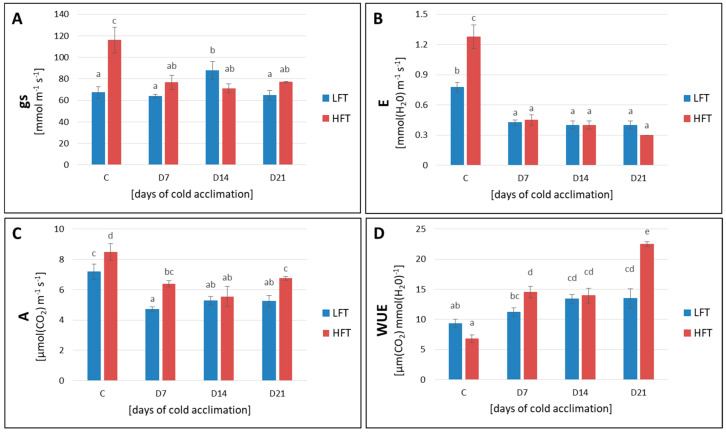
Stomatal conductance (*gs*) (**A**), transpiration rate (*E*) (**B**), assimilation of CO_2_ (*A*) (**C**), and water use efficiency (*WUE*) (**D**) in the high (HFT) and the low freezing tolerant (LFT) *Lolium multiflorum/Festuca arundinacea* introgression forms, at four time-points: before cold acclimation (CA) C and on the 7th, 14th, and 21st day of CA. Error bars represent the standard errors of four replicates. Homogeneity groups are denoted by the same letters, according to Fisher’s LSD test (*p* = 0.05).

The HFT introgression form demonstrated higher *gs*, compared with the LFT form, before CA, and this phenomenon had a reflection in higher *E* and higher *A* (Figure 3). The water use efficiency (*WUE*) revealed similar values in these two analyzed forms at this particular time-point. However, during the process of CA, *gs* remained at the same level in the LFT form, despite a temporal increase on the 14th day of CA, whereas in the HFT form a significant decrease of *gs* under CA conditions, was observed. On the other hand, a decrease of *E* and *A* parameters was noticed in both introgression forms during CA, despite differences in stomata behavior revealed between the HFT and LFT plants with respect to the conditions before CA. After the full time of CA, *A* parameter increased in the HFT form. The value of *WUE* increased under cold conditions in both introgression forms and after three weeks of CA, it was significantly higher in the HFT form (Figure 3).

### 2.4. Transcript and Protein Accumulation of pFBA and pPGK

**Figure 4:**
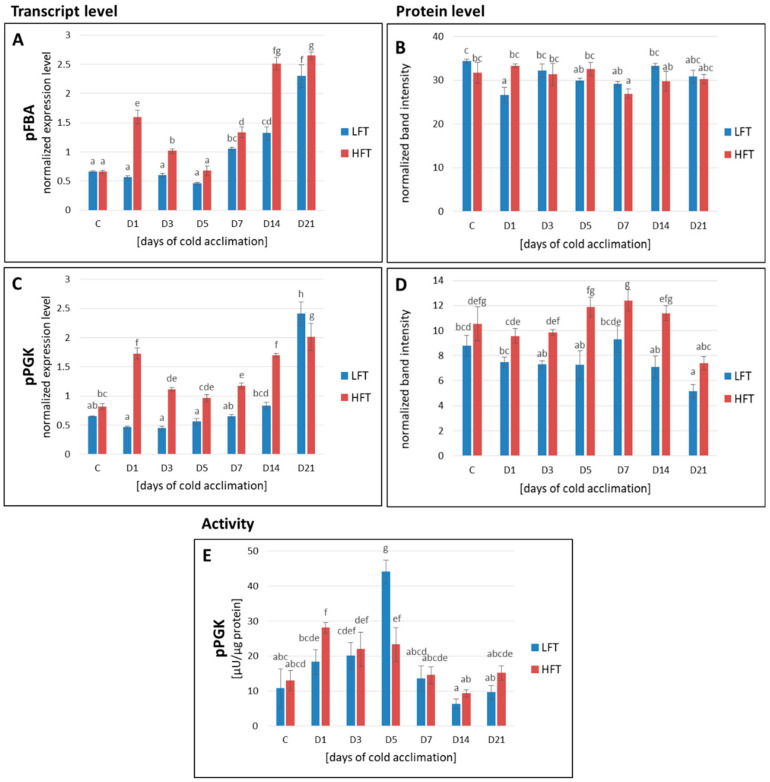
Transcript (**A**,**C**) and protein (**B**,**D**) accumulation levels of plastid fructose-1,6-bisphosphate aldolase (pFBA; (**A**,**B**)), and plastid phosphoglycerate kinase (pPGK; (**C**,**D**)), as well as the activity of pPGK (**E**), at seven time-points: before cold acclimation (CA) C and on the 1st, 3rd, 5th, 7th, 14th, and 21st day of CA in *Lolium multiflorum/Festuca arundinacea* introgression forms. Error bars represent the standard errors of three replicates for protein analyses, and four replicates for transcript analyses. Homogeneity groups are denoted by the same letters, according to Fisher’s LSD test (*p* = 0.05). HFT–high freezing tolerant introgression form, LFT–low freezing tolerant introgression form.

The transcript abundance of pFBA and pPGK showed significant differences between the analyzed introgression forms at a majority of CA time-points, and in more cases, it was higher in the HFT form. However, after three weeks of CA, abundance of pFBA transcript was higher in the HFT form, but pPGK transcript–in the LFT form (Figure 4A,C). The accumulation of pFBA protein did not reveal significant differences between particular experimental time-points, and between the analyzed introgression forms, except the 1st day of CA, with higher pFBA abundance in the HFT introgression form (Figure 4B). In contrast, the abundance of pPGK protein was significantly higher between the 3rd and the 14th day of CA in the HFT form (Figure 4D).

### 2.5. pPGK Activity

The LFT form was characterized by a higher activity of pPGK on the 5th day of CA, and the HFT form, on the 1st and 5th day of CA, compared to the conditions before CA. After five days of CA, this activity was significantly higher in the LFT, compared to the HFT introgression form (Figure 4E).

### 2.6. ROS Content

**Figure 5:**
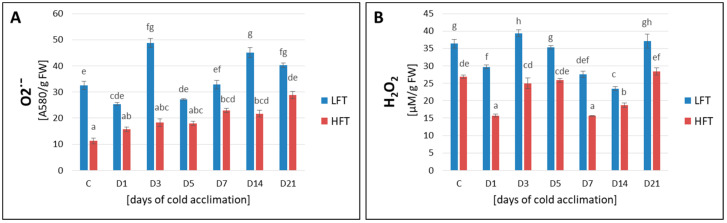
Superoxide anion radical (O_2_^•−^) (**A**), and hydrogen peroxide (H_2_O_2_) (**B**) content, at seven time-points: before cold acclimation (CA) C and on the 1st, 3rd, 5th, 7th, 14th, and 21st day of CA in *Lolium multiflorum/Festuca arundinacea* introgression forms. Error bars represent the standard errors of five replicates. Homogeneity groups are denoted by the same letters, according to Fisher’s LSD test (*p* = 0.05). HFT–high freezing tolerant introgression form, LFT–low freezing tolerant introgression form; FW–fresh weight.

The analysis of ROS content, including O_2_^•−^ (Figure 5A) and H_2_O_2_ (Figure 5B), demonstrated clear differences between the analyzed introgression forms both before and under CA conditions. The observed content of ROS was significantly higher in the LFT form at each analyzed experimental time-point. The process of CA evoked a gradual increase in O_2_^•−^ generation in the HFT form, while H_2_O_2_ remained at the level noted before CA (D3, D5, and D21) or this level was even lower (D1, D7, and D14).

### 2.7. Transcript and Protein Accumulation of APX, CAT, Fe-SOD, Mn-SOD, Cu/Zn-SOD, GR, and GPX

**Figure 6:**
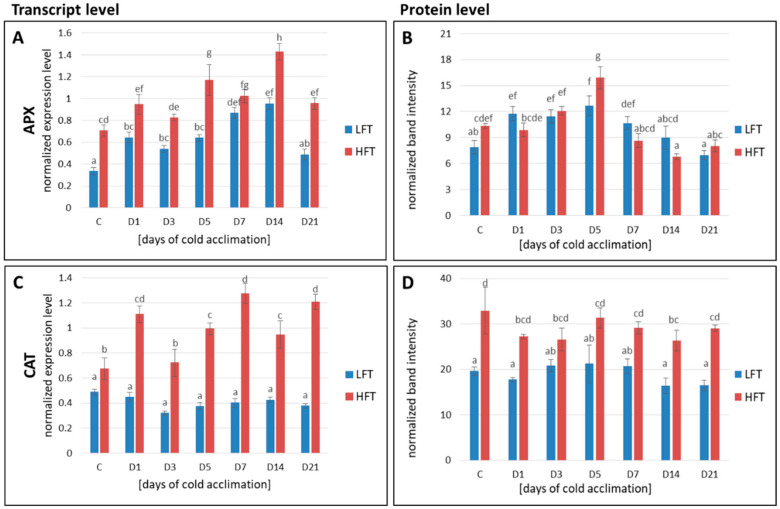

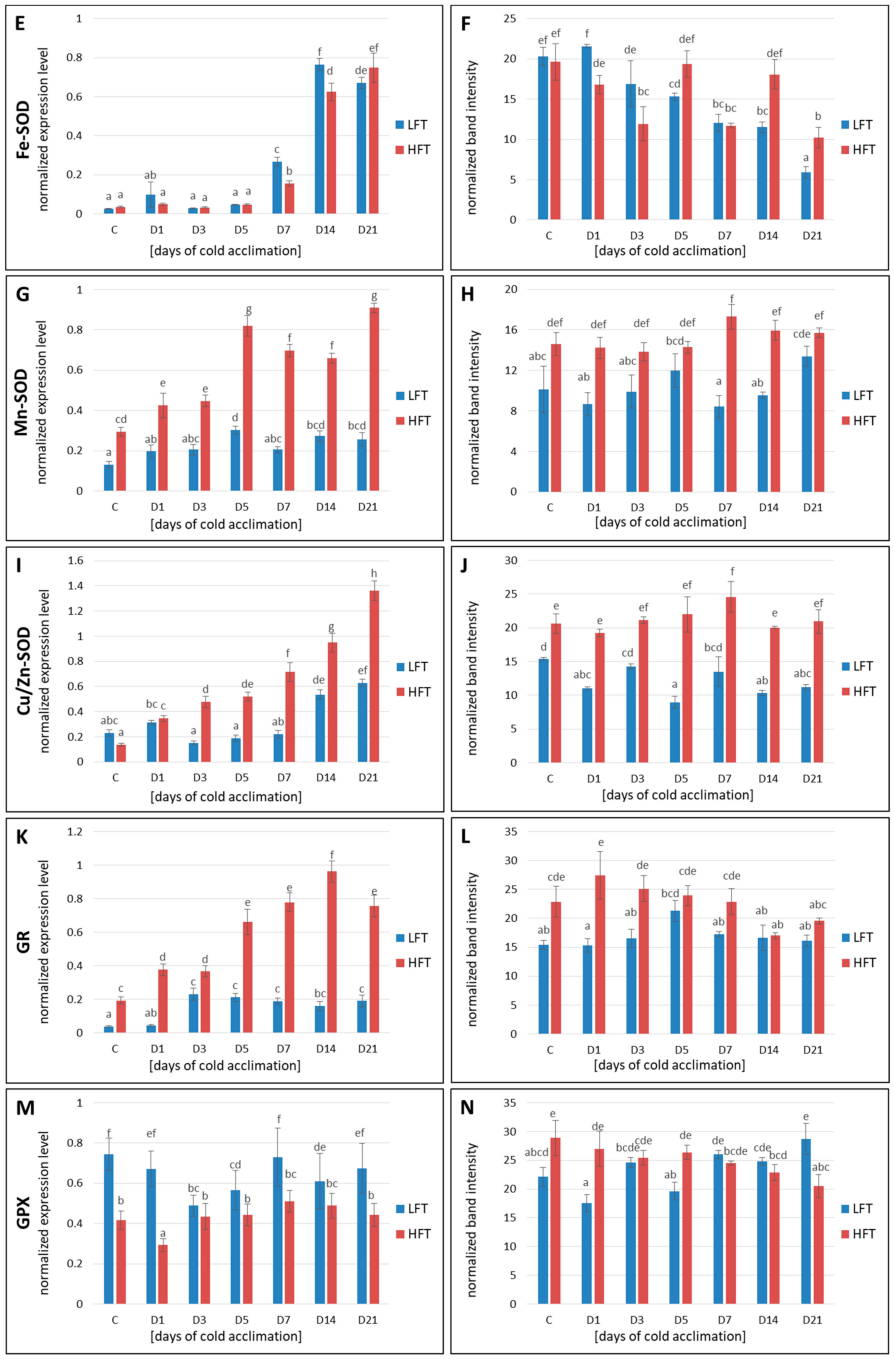
Transcript accumulation levels (**A**,**C**,**E**,**G**,I,**K**,**M**) of chloroplastic L-ascorbate peroxidase (APX) (**A**), catalase (CAT) (**C**), chloroplastic iron superoxide dismutase (Fe-SOD) (**E**), mitochondrial manganese superoxide dismutase (Mn-SOD) (**G**), chloroplastic copper/zinc superoxide dismutase (Cu/Zn-SOD) (**I**), chloroplastic glutathione reductase (GR) (**K**), chloroplastic glutathione peroxidase (GPX) (**M**); and protein accumulation levels (**B**,**D**,**F**,**H**,**J**,**L**,**N**) of chloroplastic/thylakoid L-ascorbate peroxidase (APX) (**B**), catalase (CAT) (**D**), chloroplastic iron superoxide dismutase (Fe-SOD) (**F**), mitochondrial manganese superoxide dismutase (Mn-SOD) (**H**), chloroplastic copper/zinc superoxide dismutase (Cu/Zn-SOD) (**J**), both chloroplastic and cytoplasmic glutathione reductase (GR) (**L**), chloroplastic glutathione peroxidase (GPX) (**N**); at seven time-points: before cold acclimation (CA) C and on the 1st, 3rd, 5th, 7th, 14th, and 21st day of CA in *Lolium multiflorum/Festuca arundinacea* introgression forms. Error bars represent the standard errors of three replicates for protein analyses, and six replicates for transcript analyses. Homogeneity groups are denoted by the same letters, according to Fisher’s LSD test (*p* = 0.05). HFT–high freezing tolerant introgression form, LFT–low freezing tolerant introgression form.

Higher transcript accumulation of APX, CAT, GR, Mn-SOD, and Cu/Zn-SOD was observed at a majority of analyzed time-points in the HFT form. On the other hand, transcript abundance in case of GPX was higher, except the 3rd day of CA, in the LFT form. A protein level in the case of CAT, Mn-SOD, Cu/Zn-SOD, and GR was significantly higher in the HFT introgression form at a majority of the time-points. With respect to Fe-SOD protein, its higher abundance in the HFT was mostly visible in the advanced steps of CA, while in case of APX only on the 5th day of CA. The abundance of GPX was higher in the more freezing tolerant introgression form before, and on the 1st and the 5th day of CA (Figure 6).

### 2.8. Activity of APX, POD, CAT, SOD, GR, and GPX

**Figure 7:**
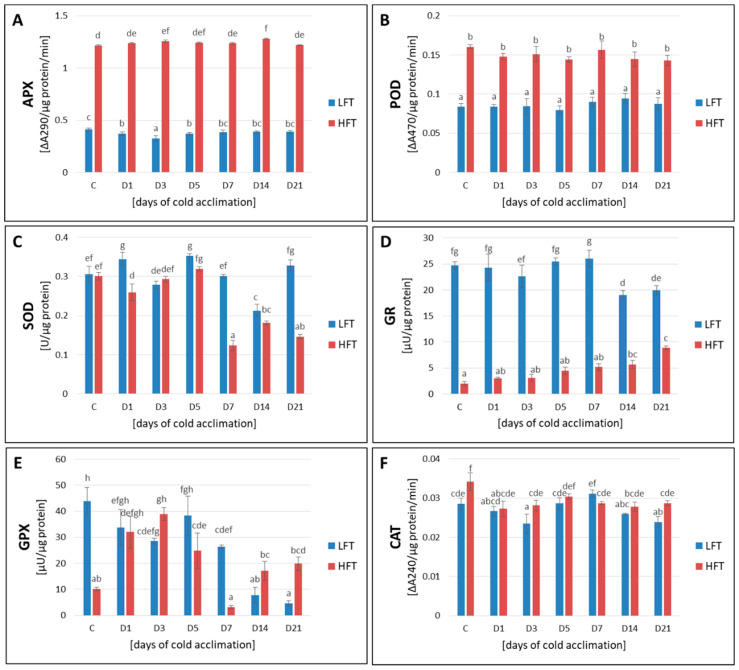
Total activity of ascorbate peroxidase (APX) (**A**), guaiacol peroxidase (POD) (**B**), superoxide dismutase (SOD) (**C**), glutathione reductase (GR) (**D**), glutathione peroxidase (GPX) (**E**), and catalase (CAT) (**F**), at seven time-points: before cold acclimation (CA) C and on the 1st, 3rd, 5th, 7th, 14th, and 21st day of CA in *Lolium multiflorum/Festuca arundinacea* introgression forms. Error bars represent the standard errors of four replicates for APX, CAT, and SOD; three replicates for GPX and GR; and six replicates for POD. Homogeneity groups are denoted by the same letters, according to Fisher’s LSD test (*p* = 0.05). HFT–high freezing tolerant introgression form, LFT–low freezing tolerant introgression form.

A higher activity of POD and APX was observed in the HFT introgression form at each analyzed experimental time-point. Additionally, after a full period of CA process, a significantly higher activity of CAT and GPX was also noticed in this form. On the other hand, the LFT form was characterized by a higher activity of GR at each experimental time-point, and by a higher total activity of SOD after the 1st, 7th, and 21st day of CA (Figure 7).

### 2.9. Accumulation of Cor14b

**Figure 8:**
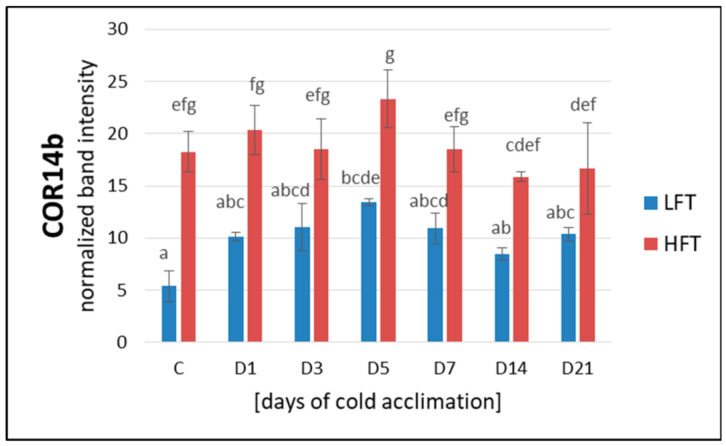
Accumulation level of Cor14b protein, at seven time-points: before cold acclimation (CA) C and on the 1st, 3rd, 5th, 7th, 14th, and 21st day of CA in *Lolium multiflorum/Festuca arundinacea* introgression forms. Error bars represent the standard errors of three replicates. Homogeneity groups are denoted by the same letters, according to Fisher’s LSD test (*p* = 0.05). HFT–high freezing tolerant introgression form, LFT–low freezing tolerant introgression form.

Accumulation of Cor14b was significantly higher in the HFT form, compared with the LFT form, over the entire experiment. However, its level in the HFT form was slightly but significantly lower after a longer period of CA (14D-21D), compared with the 5th day of CA. On the other hand, compared to the control, accumulation of this protein after five days of CA in the HFT form, was also slightly higher but statistically not significant. A quite similar accumulation dynamics was observed for the LFT form, however, on the 5th day of CA, accumulation of Cor14b was significantly higher, compared with the control conditions. No significant differences in the amount of this protein were noticed between the other experimental time-points in the LFT introgression form (Figure 8).

## 3. Discussion

Herein, we confirmed that *L. multiflorum/F. arundinacea* introgression forms could be valuable plant materials to further dissect mechanisms of CA and freezing tolerance in forage grasses. A degree of differences observed between the HFT and the LFT forms with respect to membranes’ integrity (based on T_EL50_), photoactivity (based on *Fv*/*Fm*), and gas exchange (stomatal conductance, transpiration, and CO_2_ assimilation) has changed slightly, comparing the current study and our previous work, performed on the same plants and described by Augustyniak et al. (2018) [38]. However, the physiological response to CA in the case of each analyzed introgression form has been maintained over three years, and this feature makes them good plant models to validate our scientific hypothesis set up herein but initiated already in the previous study.

### 3.1. Integrity of Cellular Membranes

Plants survival under periods of low temperature exposition depends mainly on their capacity to maintain fluidity and integrity of cellular membranes [2,42]. Water freezing inside cells always leads to cell death. Thus, a fundamental function of CA is to shift a place of water freezing from cells (cytoplasm) to intercellular spaces. Consequently, freeze-induced membrane damage results mainly from a severe dehydration associated with freezing. As temperature drops below 0 °C, ice formation is initiated in intercellular spaces and there is a movement of unfrozen water from inside the cell to the intercellular spaces [2,3]. The level of membranes’ stability, monitored as T_EL50_, is commonly used as an indicator of plant freezing tolerance, also in case of forage grasses [31,32,33,43]. Herein, the HFT introgression form revealed a higher integrity of cellular membranes, therefore also a higher level of freezing tolerance, after three weeks of CA. Though, slight differences in particular values of T_EL50_ were observed during CA process in the HFT and LFT forms between the current study and our previous research described by Augustyniak et al. (2018) [38], the general dynamics of that parameter under CA conditions was the same, and clear differences between the analyzed introgression forms continually existed. On the other hand, it is well visible that the level of freezing tolerance of the HFT and LFT forms is slightly but significantly lower in the current study, compared with the levels of tolerance observed by Augustyniak et al. (2018) [38]. Thus, we cannot exclude that the efficiency of CA in forage grasses is tightly associated with plant age. Furthermore, we cannot also exclude that now a longer period of CA would be required to achieve the same levels of freezing tolerance in the analyzed plants as those reported by Augustyniak et al. (2018) [38]. Interestingly, both introgression forms did not differ in T_EL50_ parameter on the 7th day of CA, however, this phenomenon was not due to the significant increase of freezing tolerance in the LFT form or its significant decrease in the HFT but rather to slight differences in the dynamics of CA process observed between the 5th and 7th day in the analyzed introgression forms.

The analysis of TBARS content, describing indirectly the level of lipid peroxidation, supported our data on electrolyte leakage. Lipid peroxidation affects membranes’ integrity by depletion of its fluidity, reduction of ion channels flux and inactivation of membranes’ proteins [44]. An elevated level of lipid peroxidation has been observed under numerous abiotic stresses, such as salinity, drought, cold, and exposure to heavy metals [30,45,46]. A gradual increase of TBARS content observed during CA indicated also on the elevated oxidation conditions in plant cells of both introgression forms. However, this type of stress, associated with ROS accumulation [3,47], was higher in the LFT form. On the other hand, higher susceptibility of cellular membranes to damage caused by low temperature in this form, cannot be also excluded. This phenomenon could be linked to membranes’ lipid composition [48], presence, or higher accumulation of proteins with a protective character [3], and to the accumulation of specific carbohydrates [49].

The obtained results regarding the observed levels of T_EL50_ and lipid peroxidation clearly indicated that the LFT introgression form demonstrated a significantly higher degree of cellular membranes’ damage under low temperature treatment. Cold acclimation could therefore have been a more efficient process in the HFT introgression form, as it had been indicated earlier that the key function of CA was to stabilize membranes against freezing injury [3].

### 3.2. Photosynthetic Capacity

#### 3.2.1. Photoactivity

Low temperatures affect plant photosynthetic performance and proper balance in photosynthetic capacity to maximize the available light exploitation in processes of carbon and nitrogen fixation, and to minimize harmful effects of excessively absorbed energy, should be maintained to survive this adverse environmental conditions [50,51,52,53,54]. Alterations in photoreaction centers’ excitation pressure can be directly characterized by monitoring chlorophyll fluorescence [55]. The maximum quantum yield of primary photochemistry has been proved to be a valuable marker for the performance of PSII [56]. In comparison with our previous research [38], the analyzed introgression forms in the current study demonstrated even clearer differences with respect to *Fv/Fm*. The dynamics of this parameter supported our hypothesis of better acclimation of photosynthetic apparatus to cold conditions in the HFT form. The observed phenomenon indicated, to a high probability, that protective mechanisms to prevent the possible photoinhibition, caused by low temperature and relatively high light [57], were induced in the HFT introgression form during CA. Furthermore, as *Fv*/*Fm* dynamics was maintained over several years in the plants analyzed here, it seems to be an important attribute of higher levels of freezing tolerance in *Lolium–Festuca* forage grasses. On the other hand, in the current study, *Fv*/*Fm* value did not achieve the control level after full time of CA in the HFT form, as described in our previous research by Augustyniak et al. (2018) [38], and it seems that a longer period of CA would be required to fully acclimate photosynthetic apparatus to low temperature. This phenomenon supports our conclusions regarding the level of membranes’ integrity after three weeks of CA. Also, in the case of photoactivity, plants’ age seems to be crucial for the CA process. Interestingly, similarly to T_EL50_ parameter, both introgression forms did not differ with respect to *Fv/Fm* values between the 5th and 7th day of CA. However, the strategy of photosynthetic adjustment to cold conditions, which is tightly associated with a plant capacity to acquire freezing tolerance [50,58], is a trait dependent on the existence of proper balance between energy supply and consuming (carbon metabolism reactions), as we suggested in our earlier studies on mechanisms of CA in forage grasses [33,38]. Furthermore, we assume that this trait is also strongly linked to plant potential to scavenge excess of ROS and to reduce an oxidative stress. Thus, the periodical reduction of PSII capacity in case of the HFT form, observed during the 1st week of CA, could be also associated with an overproduction of ROS and limitations in their efficient scavenging at the initial steps of CA. It has been recognized that these processes have to be carefully controlled to prevent damage of photosystems, and consequently a destruction of plant cells [57,59].

As lower photosynthetic activity may constrain CA, which is an energy consuming process [60], the plant’s ability to prevent photoinhibition seems to be strongly correlated with plant freezing tolerance. Consequently, the acclimation of photosynthetic apparatus to low temperature conditions supplies plant cells with energy required for the efficient run of CA [61]. Furthermore, Rapacz et al. (2004) [58] suggested that photosynthetic acclimation to low temperature is perhaps one of the most crucial aspects of CA in higher plants and can be, at least partially, responsible for the differential expression of freezing tolerance among different plant genotypes.

#### 3.2.2. Gas Exchange

Stomatal guard cells are parts of cellular sensing system for numerous abiotic and biotic stresses, and react rapidly triggering stomata closure under unfavorable conditions, including cold and drought [62]. At chilling temperatures, plants suffer from limited water uptake and consequently from dehydration. Thus, stomatal closure and reduced transpiration rate in response to cold can limit plant dehydration [62,63]. This inducible mechanism was noticed, e.g., in cold tolerant *Commelina communis*, but not in cold-sensitive *Nicotiana rustica* [64]. In the current study, the HFT introgression form reduced its stomatal aperture and this phenomenon was reflected by a lower transpiration rate and a lower net CO_2_ assimilation. On the other hand, the stomatal conductance of the LFT form remained at the same level before and during CA conditions, despite the 14th day of CA, whereas its transpiration rate and CO_2_ assimilation decreased significantly to the levels observed in the HFT form, indicating on non-stomatal regulations of these processes in the introgression form with a lower level of freezing tolerance. Furthermore, a lack of significant decrease of stomatal conductance during the whole experiment in the LFT introgression form, could also suggest that this form possessed, at least partially, a different strategy of low temperature sensing. After three weeks of CA, despite reduced stomatal aperture, the HFT form started to increase CO_2_ assimilation, which could be associated with the acclimation of photosynthetic apparatus to cold. However, though after a full period of CA this increase was significant, compared with the 14th day of CA, it did not reach the control values. Thus, it cannot be excluded that a longer period of CA would be required in case of the HFT form to achieve a maximum level of photosynthetic acclimation. Interestingly, the same tendency was observed in the current study also with respect to *Fv*/*Fm* parameter, which did not reach the control values after three weeks of CA in the HFT form. Contrary, in our earlier work [38], despite decreased stomatal conductance after full cold acclimation period, only slight but statistically not significant reduction of CO_2_ assimilation, was observed in the HFT form. Furthermore, also *Fv/Fm* parameter demonstrated earlier a complete turnover to the control values after three weeks of CA in the HFT form [38]. However, as gas exchange, including CO_2_ assimilation was not analyzed by Augustyniak et al. (2018) [38] after one and two weeks of CA, but only after three weeks, we could not fully understand the process of photosynthetic adjustment to low temperature in our previous research. On the other hand, as mentioned earlier, we cannot also exclude here, that some disturbances in photosynthetic acclimation to cold conditions could be associated with plants’ age, and a longer period of CA would be required in older plants to achieve full acclimation of photosynthetic apparatus. Interestingly, *WUE* revealed that the HFT introgression form exhibited a higher amount of carbon assimilated as biomass, indicating more efficient water management in this form after full time of CA, which was not observed in our earlier research [38].

#### 3.2.3. The Calvin Cycle Efficiency

It was demonstrated earlier that, during the CA of winter *Secale cereale*, photosynthesis recovered to rates similar to those revealed in non-acclimated plants at optimal growth temperatures. The authors suggested that this increase in photosynthetic activity in case of cold-hardy but not in not-hardy cereal represented an adaptive response for maintenance of basal metabolism during winter [65]. In our previous work [38], we concluded that photosynthetic acclimation to cold conditions in case of the HFT introgression form was associated with the efficiency of the Calvin cycle and that this process during CA was highly dependent on the activity of plastid fructose-1,6-bisphosphate aldolase, from the regeneration step of the cycle. Interestingly, the dynamics of aldolase activity was also parallel to the observed dynamics of *Fv*/*Fm* parameter under CA [38]. Herein, we proved that the total amount of aldolase content in cells of the analyzed introgression forms did not have a key impact on CO_2_ assimilation rate as no significant differences were observed between the HFT and LFT forms. This phenomenon supports our hypothesis that pFBA could be represented by multiple isoforms with different activities, due to different post-translation modifications in plant cells. In fact, Augustyniak et al. (2018) [38] using 2-D electrophoresis observed different aldolase isoforms between the HFT and LFT introgression forms. Thus, although total accumulation of pFBA did not show statistically significant differences between the experimental time-points and the investigated introgression forms, different isoforms of that enzyme could be accumulated at various levels in plants distinct in their freezing tolerance, thus influencing the efficiency of the Calvin cycle and the plant ability to photosynthetic acclimation to cold. In contrast, under water deficit conditions, the high drought tolerant genotypes of *F. arundinacea* and *F. glaucescens* [30], and *L. multiflorum/F. arundinacea* introgression forms, characterized by a higher photosynthetic capacity [35], revealed simultaneously both higher accumulation and activity of chloroplastic aldolase.

On the other hand, phosphoglycerate kinase from the reduction step of the Calvin cycle, revealed higher accumulation level between the 3rd and the 14th day of CA in the HFT introgression form. Our previous study on *F. pratensis* showed that during CA, pPGK was highly accumulated in the HFT genotype [33]. Interestingly, also under water deficit conditions, higher accumulation of pPGK was noticed at numerous time-points of drought period in the high drought tolerant genotypes of *F. arundinacea* and *F. glaucescens* [30]. However, in the current study, this phenomenon was not followed by the pattern of pPGK activity, which was even temporary higher on the 5th day of CA in the LDT form. Unfortunately, the activity of pPGK was not analyzed in our earlier research [30,33]. On the other hand, this temporal increase of activity revealed here, did have no impact on the level of CO_2_ assimilation in the LFT form, which was shown to be stable during CA process in case of this introgression form. Thus, it is highly probable that the regeneration step of the Calvin cycle is the most important component of dark photosynthetic phase, involved in the process of photosynthetic acclimation to low temperature in *L. multiflorum/F. arundinacea* introgression forms. We cannot also exclude that the HFT and the LFT plants accumulated the enzymatic isoforms of p PGK with different activities, similarly to pFBA. However, this aspect of our study requires further research.

### 3.3. ROS Content and Antioxidant Capacity

Overproduction of ROS and disturbances in cellular redox homeostasis leads to metabolic dysfunctions in plant cells and in certain circumstances, also to cell death. On the other hand, ROS act as effector molecules in numerous biological processes, associated with cell signalling, differentiation, growth and proliferation, reactions to external stimuli, as well as programmed cell death [11,12,17,19,66,67,68]. Reactive oxygen species emerged along with aerobic life origination and accompanied our existence to the present day [69]. Therefore, environmental factors, both biotic and abiotic, affecting photosynthetic and photorespiration rates, can have a negative impact on redox balance, and an excess of ROS can damage cellular components, including nucleic acids, proteins, and lipids [12,19,70,71]. Due to harmful effects of overproduced ROS, plants developed an antioxidant system, responsible for scavenging their excess. The enhanced performance of antioxidant system during cold stress has been well documented in Arabidopsis by Juszczak et al. (2016) [72].

As metabolisms of O_2_^•−^ and H_2_O_2_ are tightly associated [12,19], a strong relationship between the accumulation levels of these particles, has been observed. This phenomenon was also revealed in our study. Furthermore, as a periodic decrease of H_2_O_2_ content in the HFT form under low temperature stress, was also noticed, it cannot be excluded that these unfavorable conditions could create specific redox balance in plant cells, which consequently could promote the activation of some tolerance-related genes to improve freezing tolerance of the HFT form. Hydrogen peroxide was proved earlier to be involved in a regulation of gene expression [12,19]. Generally, a significantly lower content of analyzed ROS in the more freezing tolerant introgression form suggests, first of all, that the acclimation of photosynthetic apparatus to cold conditions in this form, and a lower energy imbalance, could be an important attribute of the HFT introgression form in cellular mechanisms to reduce a production of higher amounts of ROS. On the other hand, the antioxidant machinery in the HFT form could exhibit more effective scavenging of ROS, to protect plants against cell damage, manifested here also by lower membrane lipid peroxidation.

A lower level of H_2_O_2_ content in the HFT introgression form was, to a high probability, associated with a relatively high activity of POD and APX, during the whole experimental period, including both the conditions before and during acclimation. Furthermore, higher activities of CAT and GPX, especially at the advanced time-points of CA, seem to be involved in this phenomenon. Low temperature was proved to increase the activity of SOD and CAT in *Hordeum vulgare*, *S. cereale, Triticum aestivum,* and *T. durum* [73]. Furthermore, in the other research on *T. aestivum* it was shown that low temperature increased the activity of SOD, APX, GR, and POD [74]. Interestingly, in the HFT form, higher accumulation of CAT transcript and protein at each analyzed time-point, and higher accumulation of APX transcript at a majority of experimental time-points, were observed. This phenomenon indicates on a relatively high potential of this introgression form to reduce H_2_O_2_ content and to maintain the balanced capacity of antioxidant system by incessant synthesis of its components to compensate the instability of enzymes. A positive impact on ROS scavenging and a lower level of chlorophyll degradation in *F. arundinacea*, overexpressing APX and Cu/Zn-SOD, was observed by Lee et al. (2007) [75]. Better tolerance of low temperature was also noticed in *Lycopersicon esculentum*, overexpressing tonoplast APX. Its higher accumulation level was shown to be associated with a decreased photoinhibition [76].

On the other hand, although the HFT introgression form revealed generally a significantly lower content of O_2_^•−^ at each analyzed time-point of the performed experiment, the profile of SOD activity did not follow *per se* this tendency. First of all, a lack of such the directed relationship between both parameters could be, in our opinion at least partially, attributed to the fact that a total SOD activity was measured in this study, but activities of particular SOD isoforms could have been more responsible for O_2_^•−^ content. It is highly probable, since transcript and protein accumulation levels of Mn-SOD and Cu/Zn-SOD were higher at a majority of analyzed experimental time-points in the HFT form. Furthermore, this tendency was also well-visible at the advanced CA period in case of Fe-SOD protein. On the other hand, as metabolisms of O_2_^•−^ and H_2_O_2_ are tightly associated, a relatively low activity of SOD could be required to maintain a low level of H_2_O_2_ content. By maintaining the stable level of H_2_O_2_ and the level of free transient metals such as Fe^2+^ under control, plant cells are able to prevent the formation of highly toxic hydroxyl radical via the Fenton reaction, and consequently to reduce DNA damage, protein oxidation and lipid peroxidation [19,77]. Finally, O_2_^•−^ can also be used by nitric oxide for the production of peroxynitrite, which is a strong oxidant and nitrating molecule. Nitric oxide competes with SOD for available O_2_^•−^ molecules, what could be responsible for its lower level in plant cells of the HFT form [78]. Additionally, peroxynitrite can be inactivated by GPX via its reduction [79]. Thus, a higher accumulation of GPX protein at several analyzed time-points together with its higher activity in fully acclimated the HFT form, could also be responsible for the metabolism of reactive oxygen and nitrogen species in these plants. A lack of positive relation between accumulation and activity of GR, observed in the analyzed introgression forms, might depend on the level of GSH. Although GR activity was relatively low in the HFT form, compared with the LFT form, it increased significantly under stress conditions, which was most likely related to a high abundance of GSH in cells of this introgression form.

### 3.4. Cor14b Accumulation and Cellular Protection against Photoinhibition

A large number of genes have been shown to be triggered by chilling conditions [3,80,81,82]. Plenty of them are known to encode proteins involved in the acquisition of freezing tolerance. Cold-regulated genes constitute a family with large numbers of genes rapidly induced by low temperature during CA process [3]. *Cor14b* gene is one of the most extensively characterized members of *Cor* family in monocotyledons. The level of its expression was shown to be associated directly with a combined tolerance to photoinhibition and freezing [9,83,84]. It has been shown that light and temperature changes both had an impact on the CA process [85]. Chloroplasts were proved to play active roles not only as photosynthetic organelles, but also as sensors of environmental stresses [50,86,87]. The accumulation of Cor14b protein was promoted directly by chloroplast redox state, while a level of *cor14b* transcript increased after cold treatment exclusively [84]. The direct role of Cor14b protein in cellular mechanisms to prevent freezing damage has not been recognized in detail. However, to date it has been demonstrated in *H. vulgare* that this protein was accumulated in chloroplastic stroma, and could be involved in the protection of plant cells against photoinhibition [9,88]. In our study, the accumulation of Cor14b protein was significantly higher in the HFT form during the whole experimental conditions, including both the periods before and under CA conditions. A relatively high content of Cor14b on the last day of pre-hardening in both analyzed introgression forms, was probably due to the fact that its accumulation could have been initiated already under pre-hardening period, when a relatively lower temperature (12 °C) was applied. Differences observed with respect to the levels of Cor14b accumulation between the introgression forms with distinct levels of freezing tolerance could be responsible, at least partially, for different capacities of the analyzed plants to acclimate their photosynthetic apparatus to cold conditions under both pre-hardening and CA periods, and to prevent further photoinhibition, as suggested earlier by Rapacz et al. (2008) [9]. It has been proved earlier that Cor15a, an analog of Cor14b, was involved in stabilizing membranes’ integrity under low temperature in Arabidopsis [40,41]. Thus, the impact of Cor14b accumulation on the stability of thylakoid membranes during CA in *L. multiflorum/F. arundinacea* introgression forms cannot be also excluded. A preservation of cellular membrane integrity, as a primary site of freezing injury, is a priority for plant survival under prolonged exposure to low temperature [3].

## 4. Materials and Methods

### 4.1. Plant Materials and Experimental Set Up

Plant materials used in the research involved two tetraploid introgression forms of *L. multiflorum/F. arundinacea* contrasting in their freezing tolerance, the HFT and LFT form. Both introgression forms were obtained after five rounds of backcrossing of partially fertile *F. arundinacea* (6*x*) × *L. multiflorum* (4*x*) F_1_ hybrid (2*n* = 5*x* = 35) to tetraploid cultivars of *L. multiflorum* [38]. The freezing tolerance of these two introgression forms was evaluated earlier (in 2015) on the basis of (i) their ability to regrow after freezing, using the method described by Larsen (1978) [89], and (ii) stability of their cellular membranes after freezing, applying measurements of T_EL50_ [38]. Furthermore, also the other physiological parameters precisely describing plant functioning under cold acclimation conditions, including chlorophyll fluorescence and gas exchange, were analyzed earlier for the HFT and LFT introgression forms [38]. However, as all these parameters are crucial to estimate plant physiological performance under particular experimental conditions, especially following three years of plant vegetation, we decided to analyze T_EL50_, chlorophyll fluorescence and gas exchange also under current study in the same experimental conditions (in 2018) to be sure that both introgression forms still possess the physiological traits characterized and described earlier by Augustyniak et al. (2018) [38].

**Figure 9:**
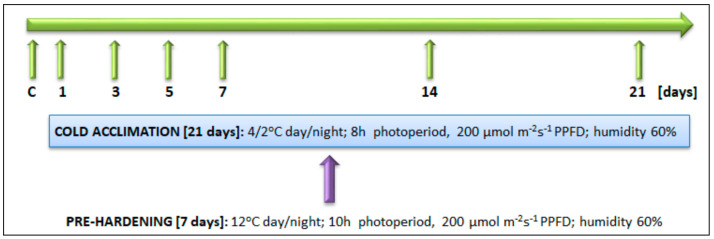
The scheme illustrating the experimental set up.

Each introgression form was multiplicated in 10 independent clones (each one planted in individual pot measuring 1.75 dm^3^, containing a sand:peat (1:1) mixture). Each clone represented a single biological replicate of the particular introgression form. The details concerning plant propagation have been already described in our previous papers [32,33]. The experiments involved two separate environmental conditions: (i) pre-hardening, 7 days of 12 °C, 10 h photoperiod, 200 μmol^−2^ s^−1^ photosynthetic photon flux density, PPFD and humidity 60% and (ii) CA, 21 days of 4/2 °C day/night, 8 h photoperiod, 200 μmol^−2^ s^−1^ PPFD and humidity 60%, as described by Augustyniak et al. (2018) [38]. The measurements of T_EL50_, chlorophyll fluorescence, gas exchange, ROS and TBARS contents, were performed using the 2nd, fully expanded and undamaged leaves. The analysis of transcript and protein accumulation, as well as enzymatic activities of the Calvin cycle and antioxidant proteins, were performed using pooled leaves. All the measurements were performed at the defined experimental time points (at the 2nd hour of photoperiod), as described earlier by Augustyniak et al. (2018) [38] and presented in the Figure 9.

### 4.2. Membrane Integrity

#### 4.2.1. Temperature Causing a 50% Electrolyte Leakage (T_EL50_)

To determine T_EL50_ (for each freezing temperature: from −2 to −18 °C range), the leaves were cut in five replicates of each introgression form, divided into 2-cm-long segments, and placed on ice (5 cm^3^ of frozen deionized water) to ensure ice nucleation in conductivity vessels. The vessels were then put into a programmed low temperature thermostat (Lauda E200) with the temperature of 0 ± 0.5 °C. A freezing-thawing cycle was performed in darkness, separately for each freezing temperature. The temperature was decreased at a rate of 2 °C h^−1^. Freezing temperature was kept for 90 min and then temperature was increased up to 0 °C at a rate of 3 °C h^−1^. The membranes’ injuries after freezing were determined by measuring the electrical conductivity of tissue extracts, described in detail by Rapacz (1999) [90]. The index of injuries was calculated from a linear regression fitted to the central (linear) part of the sigmoid relationship between the freezing temperature and the electrolyte leakage, using at least three temperatures. The measurements were performed one day before CA initiation and on the 1st, 3rd, 5th, 7th, 14th, and 21st day of CA [38].

#### 4.2.2. Lipid Peroxidation

The level of lipid peroxidation was estimated as the amount of TBARS according to the method of Heath and Packer (1968) [91]. Fresh leaf samples (0.3 g) were ground in 0.25% thiobarbituric acid (TBA) in 10% trichloroacetic acid (TCA) using mortar and pestle. The mixture was heated at 100 °C for 15 min and then cooled in ice bath and centrifuged at 10,000× *g* for 10 min. The absorbance of supernatant was measured at 532 nm and corrected for a nonspecific absorption by subtracting absorbance read at 600 nm. The measurements were performed with the Ultrospec 1100 pro reader (Amersham Biosciences, Chalfont St. Giles, UK). A reagent blank was run simultaneously and consisted of the extract treated only with 10% TCA. The concentration of TBARS was calculated using its absorption coefficient λ = 155 mM^−1^ cm^−1^. Results are shown in μmol per 1 g fresh weight. The measurements were carried out in five replicates at each time-point.

### 4.3. Capacity of Photosynthetic Apparatus

#### 4.3.1. Chlorophyll Fluorescence

Chlorophyll fluorescence was measured by using the HandyPEA fluorimeter (Hansatech Instruments Ltd., Kings Lynn, UK). Before measurements, the internal LED-light source was calibrated using an SQS light meter (Hansatech Instruments Ltd., Kings Lynn, UK). Measurements were taken with the saturated excitation light of 3.000 μmol (quanta) m^−2^ s^−1^ after 30 min of adaptation in the dark, in leaf clips (Hansatech Instruments Ltd., Kings Lynn, UK). Changes in the chlorophyll fluorescence signal were registered between 10 μs and 1 s of saturated light pulse. During the initial 2 ms, data was collected every 10 μs with a 12-bit resolution. After this period, the frequency of measurements was reduced automatically. The fast kinetics of chlorophyll a fluorescence (OJIP test) parameters were calculated using the generated chlorophyll fluorescence induction curve [92,93]. The measurements were carried out in ten replicates at each time-point.

#### 4.3.2. Gas Exchange

Assimilation of CO_2_, transpiration and stomatal conductance were measured using an infrared gas analyzer (Ciras-3, PP Systems, Hitchin, UK), with a Parkinson leaf chamber (PLC6) automatically controlling the measurement conditions (irradiance = 500 μmol (quanta) m^−2^ s^−1^, halogen lamps, RH = 30%, CO_2_ concentration (400 μmol (CO_2_) mol^−1^ (air)). The airflow rate through the assimilation chamber was 350–400 cm^3^ min^−1^. The measurements were made in the middle part of leaves and were performed for four replicates of each introgression form on one day before cold acclimation initiation and on the 7th, 14th, and 21st day of CA, in the temperature designed for these time-points according to Augustyniak et al. (2018) [38]. Based on the measured gas exchange parameters, water use efficiency was calculated as the ratio of the rate of CO_2_ assimilation to the rate of transpiration.

#### 4.3.3. Transcript Accumulation-RT-qPCR (pFBA, EC 4.1.2.13 and pPGK, EC 2.7.2.3)

Total RNA was extracted from leaves of the analyzed plants, using RNeasy Plant Mini Kit (Qiagen, Hilden, Germany), and then treated with DNAse I (Roche, Basel, Switzerland). Afterwards, cDNA was synthesized from 1 μg of RNA using Maxima H Minus First Strand cDNA Synthesis Kit (Thermo scientific, Waltham, MA, USA), as recommended by the manufacturer. Reverse transcription quantitative polymerase chain reaction (RT-qPCR) analysis was performed using Bio-Rad CFX 96 thermal system (Hercules, CA, USA).

A reaction mixture of final 10 μL volume contained 500 nmol of each primer, 1 μL of cDNA, and 5 μL of FastStart Essential DNA Probes Master (Roche, Basel, Switzerland). All the PCR reactions were performed using the same set of parameters: 95 °C for 10 min, 44 cycles of 95 °C for 15 s, and 60 °C for 30 s. For *real-time* reactions, a normalization with actin as a reference gene, was made. The unique primers and TaqMan probe sequences for *real time* RT-qPCR were designed using Beacon designer software, as described in our different work [30]. The sequences of primers and TaqMan probe for particular genes, are shown in Table 1. The measurements were carried out in four replicates at each time-point.

#### 4.3.4. Protein Accumulation-Western Blot (pFBA and pPGK)

The accumulation levels of investigated here enzymes of the Calvin cycle were estimated by use of Western blot analysis with specific antibodies. All the antibodies were produced earlier by Agrisera^®^ company (Vännäs, Sweden) using a rabbit host immunized with a highly specific amino acid peptides: pFBA (TFEVAQKVWAETFYY), as described previously in Perlikowski et al. (2016) [35], and pPGK (GITVTKADDVIGPEC), as described by Lechowicz et al. (2020) [30]. Protein extraction and Western blotting procedures were performed as described by Pawłowicz et al. (2012) [94]. Briefly, 10 µg of total proteins were separated in 12% SDS-polyacrylamide gel electrophoresis and electroblotted onto nitrocellulose membranes (Amersham™ Protran™ Premium 0.2 µm NC). Target proteins were detected using appropriately matched and labeled rabbit polyclonal antibodies (anti-pPGK and anti-pFBA) diluted 1:4000. The antigen–antibody complexes were detected using a secondary anti-rabbit IgG–horseradish peroxidase conjugate (diluted 1:20000; Sigma, St. Louis, MO, USA, currently member of Merck Group, Dormstadt, Germany), chemiluminescent substrate (Westar Supernova–Cyanagen, Bologna, Italy), and ChemiDoc™ Touch Igmagin System (Bio-Rad, Hercules, CA, USA). The intensities of visualized bands were estimated using ImageJ software. To normalize the measurements of protein band intensities between the different Western blots, the measurements from a single membrane were divided by the mean of two standard samples separated on this blot and further multiplied by the mean of standards from all the blots. Normalization was performed to minimize the differences that occurred between blots during the separated procedures. The measurements were carried out in three replicates at each time-point.

#### 4.3.5. pPGK Activity

The activity of pPGK was evaluated using Phosphoglycerate Kinase Activity Assay Kit (Colorimetric) (ab252890-abcam^®^ company, Cambridge, UK), following the instructions attached by the manufacturer. Protein extracts from chloroplasts were prepared according to a modified method described in detail by Kosmala et al. (2012) [95]. Briefly, 1g of frozen leaf material was ground in a liquid nitrogen, suspended in 4 mL of chloroplast isolation buffer (Sigma, St. Louis, MO, USA, currently member of Merck Group, Dormstadt, Germany), shaken, filtered through a mesh 100 nylon and centrifuging 3 min at 200× *g* at 5 °C. The collected supernatant was subsequently centrifuged for 15 min at 900× *g* at 5 °C, and the washed chloroplast pellet was suspended in 150 μL 0.1 M phosphate buffer (0.1 M Na_2_HPO_4_) with 3% Triton X100, shaken 5 min in 1000 rpm, and centrifuged for 15 min at 13,000× *g*. Then, 50 μL of collected supernatant (enzyme extract) was used to determine the pPGK activity. One unit of pPGK was defined as the amount of enzyme that generates 1.0 µmol of 1,3-bisphosphoglycerate per min at pH 7.2 at 37 °C. Activity measurements were performed with the Synergy HTX Multi-Mode Reader (BioTek, Winooski, VT, USA). The measurements were carried out in three replicates at each time-point. The values obtained for each enzymatic activity were normalized using the soluble protein concentration and expressed per 1 μg of protein. Protein concentration was determined according to Bradford (1976) [96].

### 4.4. ROS Content and Capacity of Antioxidant System

#### 4.4.1. ROS Content

A content of hydrogen peroxide (H_2_O_2_) was measured spectrophotometrically using the titanium (Ti^4+^) method described by Becana et al. (1986) [97]. Fresh leaves (0.25 g) were homogenized in 1.5 mL of 0.1 M potassium phosphate buffer (pH 7.8). After centrifugation (15,000× *g* for 30 min), supernatant was used for further assays. The reaction mixture (1.5 mL) contained 0.1 M potassium phosphate buffer (pH 7.8), enzymatic extract (400 μL) and titanium reagent. Titanium reagent was prepared on the day of assay by mixing 0.6 mM solution of 4-(2-pyridylazo) resorcinol and 0.6 mM potassium titanium tartrate at a 1:1 ratio. The prepared solution was kept in ice bath. The concentration of H_2_O_2_ was estimated by measuring absorbance at a wavelength of 508 nm, against a calibration curve and expressed as μmol H_2_O_2_ per 1 g fresh weight (FW). The measurements were carried out in five replicates at each time-point. A content of superoxide anion (O_2_^•−^) was assayed spectrophotometrically on the basis of the capacity of superoxide anion radical to reduce nitrobluetetrazolium (NBT) to diformazan [98]. Twelve leaves discs from (0.35 cm in diameter) were incubated with 3 mL of a mixture containing 0.05 M potassium-phosphate buffer (pH 7.8) with 0.1 mM EDTA, 10 mM NaN_3_, and 0.05% NBT, for 1 h in the dark. Next, samples were heated 15 min at 85 °C and cooled down on ice. The absorbance was measured at wavelength of 580 nm. The level of O_2_^•−^ was determined from the absorbance change at 580 nm per 1 g of FW. The measurements were carried out in five replicates at each time-point.

#### 4.4.2. Transcript Accumulation

RT-qPCR (chloroplastic L-ascorbate peroxidase (APX, EC 1.11.1.11), catalase (CAT, EC 1.11.1.6), chloroplastic iron superoxide dismutase (Fe-SOD, EC 1.15.1.1), mitochondrial manganese superoxide dismutase (Mn-SOD, EC 1.15.1.1), chloroplastic copper/zinc superoxide dismutase (Cu/Zn-SOD, EC 1.15.1.1), chloroplastic glutathione reductase (GR, EC 1.6.4.2), and chloroplastic glutathione peroxidase (GPX, EC 1.11.1.9).

Total RNA extraction, cDNA synthesis, and RT-qPCR reactions were performed as described in Section 4.3.3. The unique primers and TaqMan probe sequences for *real time* RT-qPCR were designed using Beacon designer software, as described in our different work [30]. The sequences of primers and TaqMan probe for particular genes, are shown in Table 2. The measurements were carried out in six replicates at each time-point.

#### 4.4.3. Protein Accumulation-Western blot (Chloroplastic/Thylakoid APX, CAT, Chloroplastic Fe-SOD, Mitochondrial Mn-SOD, Chloroplastic Cu/Zn-SOD, Chloroplastic and Cytoplasmic GR, and Chloroplastic GPX)

The procedure of protein extraction, electrophoresis, electrotransfer, and Western blot were the same as described in Section 4.3.4. The antibodies directed against particular enzymes were commercially available and produced by Agrisera^®^ company (Vännäs, Sweden): APX (AS08368), CAT (AS09501), Fe-SOD (AS06125), Mn-SOD (AS09524), Cu/Zn-SOD (AS10652), GR (AS06181), and GPX (AS04055). The primary antibody was diluted 1:4000, and the antigen–antibody complexes were detected using a secondary anti-rabbit IgG–horseradish peroxidase conjugate diluted 1:20000 (Sigma, St. Louis, MO, USA, currently member of Merck Group, Dormstadt, Germany). The measurements were carried out in three replicates at each time-point.

#### 4.4.4. Antioxidant Enzymes Activity

The values obtained for each enzymatic activity were normalized using the soluble protein concentration and expressed per 1 μg of protein. Protein concentration was determined according to Bradford (1976) [96]. A total activity of investigated enzymes was analyzed.

##### Ascorbate Peroxidase

Ascorbate peroxidase activity was assayed using a method modified after Nakano & Asada (1981) [99], as described in detail by Arasimowicz-Jelonek et al. (2011) [100]. APX activity was determined following protein extraction from 0.5 g tissue homogenized in 2 mL 50 mM potassium phosphate buffer (pH 7.8). The reaction mixture contained 1.2 mL 0.1 M potassium phosphate buffer (pH 7.0), 2.6 mM sodium ascorbate, 48 mM H_2_O_2_ and 300 µL of enzyme extract. The activity of APX was determined from the absorbance change at 290 nm min^−1^. Activity measurements were performed with the Ultrospec 1100 pro reader (Amersham Biosciences, Chalfont St. Giles, UK). The measurements were carried out in four replicates at each time-point.

##### Catalase

Catalase activity was determined spectrophotometrically, according to Dhindsa et al. (1981) [101], detailed outline by Arasimowicz-Jelonek et al. (2011) [100]. CAT activity was determined following protein extraction from 0.1 g tissue homogenized in 2 mL 0.1 M sodium phosphate buffer (pH 7.0). The reaction mixture (3 mL) contained 0.1 M sodium phosphate buffer (pH 7.0), 100 µL of enzyme extract, and 3% H_2_O_2_. The activity of CAT was determined from the absorbance change at 240 nm based on the amount of decomposed H_2_O_2_. One unit of CAT activity was defined as the amount of enzyme catalyzing a decomposition of 1 μmol H_2_O_2_ min^−1^ calculated from the extinction coefficient 45.2 mM^−1^ cm^−1^. Activity measurements were performed with the Ultrospec 1100 pro reader (Amersham Biosciences, Chalfont St. Giles, UK). The measurements were carried out in four replicates at each time-point.

##### Superoxide Dismutase

Superoxide dismutase activity was evaluated according to Beauchamp & Fridovich (1971) [102], with modifications. An enzymatic extract was obtained following protein extraction from 0.1 g tissue homogenized in 2 mL of 0.1 M sodium phosphate buffer (pH 7.0) containing 1 mM EDTA, 1% PVP and 0.01 M NaCl. The reaction mixture (3 mL) contained 0.1 M sodium phosphate buffer (pH 7.0), 13 mM of methionine, 75 µM of NBT, 20 µL of enzyme extract and 2 µM of riboflavin. Addition of riboflavin initiated the reaction. The reaction mixture was exposed to UV light (15 W) for 15 min. Absorbance was measured at 560 nm. An inhibition of reduction of water-soluble tetrazolium salt (WST-1) which produces water-soluble formazan dye upon reduction by superoxide anion radicals, which are generated by xanthine oxidase and inhibited by SOD, was measured. One unit was defined as the amount of enzyme required to cause 50% inhibition of substrate reduction under assay conditions. Activity measurements were performed with the Ultrospec 1100 pro reader (Amersham Biosciences, Chalfont St. Giles, UK). The measurements were carried out in four replicates at each time-point.

##### Glutathione Peroxidase

The activity of glutathione peroxidase was determined using Glutathione Peroxidase Assay Kit (Colorimetric) (ab102530-abcam^®^ company, Cambridge, UK), according to the enclosed protocol. The amount of 0.1 g leaf tissue was homogenized in 0.2 mL ice cold assay buffer, and 25 μL of enzyme extract was taken to the analysis. One unit was defined as the amount of enzyme which causes the oxidation of 1.0 µmol of NADPH to NADP^+^ under the assay kit condition min^−1^ at 25 °C. Activity measurements were performed with the Synergy HTX Multi-Mode Reader (BioTek, Winooski, VT, USA). The measurements were carried out in three replicates at each time-point.

##### Glutathione Reductase

The activity of glutathione reductase was evaluated using Glutathione Reductase Assay Kit (Colorimetric) (ab83461-abcam^®^ company, Cambridge, UK), following the instructions attached by the manufacturer. The amount of 0.1 g leaf tissue was homogenized in 0.5 mL cold assay buffer, and 50 μL of enzyme extract was taken to the analysis. One unit was defined as the amount of enzyme that generates 1.0 μmol of TNB min^−1^ at 25 °C. Activity measurements were performed with the Synergy HTX Multi-Mode Reader (BioTek, Winooski, VT, USA). The measurements were carried out in three replicates at each time-point.

##### Guaiacol Peroxidase (EC 1.11.1.7)

The activity of POD was determined in fresh leaves (0.25 g) homogenized in ice cold 50 mM potassium-phosphate buffer (pH 7.8) (1:4; *w*/*v*), then centrifuged at 15,000× *g* for 30 min. The reaction mixture (1.5 mL) contained 0.025 M potassium phosphate buffer (pH 7.8), 0.1 M H_2_O_2_, 20µL of enzyme extract, and 0.25% guaiacol. Application of guaiacol initiated the reaction, which was held at 20 °C. Absorbance was measured at 470 nm. POD actvity was expressed as the absorbance change at 470 nm min^−1^. Activity measurements were performed with the Ultrospec 1100 *pro* reader (Amersham Biosciences, Chalfont St. Giles, UK). The measurements were carried out in six replicates at each time-point.

### 4.5. Cor14b

#### 4.5.1. Cloning of *Cor14b* cDNA

The full-length cDNA sequence encoding protein Cor14b was obtained from the genome of *L. multiflorum/F. arundinacea* introgression forms using PCR method. Primers for the protein coding region were designed based on *L. multiflorum* mRNA sequence, obtained by screening *L. multiflorum* mRNA database (https://www.ncbi.nlm.nih.gov/nuccore/GBXX00000000) [103] with *H. vulgare cor14b* sequence [gi|24528339|emb|AJ512944.1]. The resulting PCR product was purified using QIAEXII Gel Extraction Kit (Qiagen, Hilden, Germany) and ligated into the pGEM-T Easy vector (Promega, Madison, WI, USA). Next, *Escherichia coli* strain XL1 Blue was transformed with the ligation mixture. The selected X-Gal and IPTG clones carrying an appropriate PCR product were sequenced (Molecular Biology Techniques Laboratory, Faculty of Biology, Adam Mickiewicz University, Poznań, Poland). The obtained sequences were processed with BioEdit software (ver 7.2.5).

#### 4.5.2. Protein Accumulation-Western Blot (Cor14b)

The procedure of protein extraction, electrophoresis, electrotransfer, and Western blot were the same as described in Section 4.3.4. The target protein was detected using a specific polyclonal antibody produced by Agrisera^®^ company (Vännäs, Sweden), using a rabbit host, immunized with a highly specific amino acid peptide–GKAQEATEGAVEGAKTC. The peptide was designed using amino acid sequence of Cor14b obtained on the base of the cloned cDNA of *cor14b*. The primary antibody was diluted 1:5000, and the antigen–antibody complexes were detected using a secondary anti-rabbit IgG–horseradish peroxidase conjugate diluted 1:20,000 (Sigma, St. Louis, MO, USA, currently member of Merck Group, Dormstadt, Germany). To normalize the measurements of protein band intensities between the different Western blots, the measurements from a single membrane were divided by the mean of two standard samples separated on this blot and further multiplied by the mean of standards from all the blots. Normalization was performed to minimize the differences occurred between blots during the separated procedures. The measurements were carried out in three replicates at each time-point.

### 4.6. Statistical Analysis

All the experiments included at least three independent biological replicates. For each experiment, means of the obtained values were calculated along with standard errors. Using two-way ANOVA analysis with genotype and treatment as classification factors, Fisher’s least significant difference (LSD) was made using STATISTICA10 software (StatSoft, Tulsa, OK, USA). The significant effects of genotype, time, and genotype × time interaction were selected using the family-wise error rate less than 5%.

## 5. Conclusions

**Figure 10:**
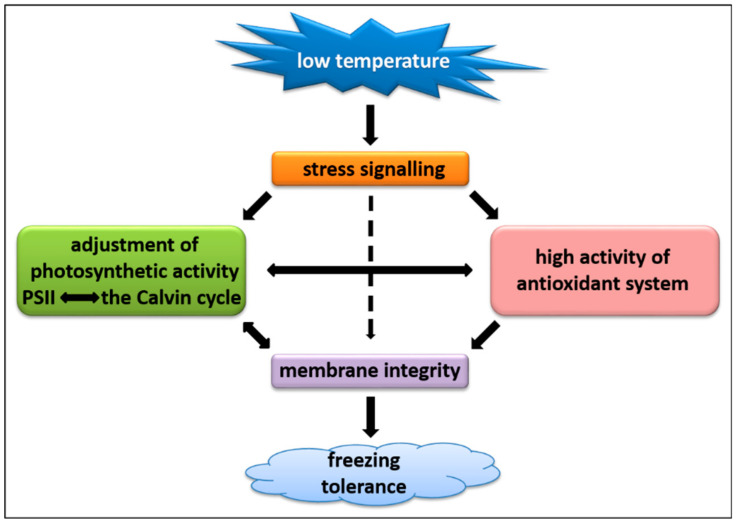
A hypothetical model demonstrating a part of cellular reactions to low temperature, including antioxidant and photosynthetic activities in cells of analyzed *Lolium multiflorum/Festuca arundinacea* introgression forms; PSII–photosystem II.

In the current study, we revealed a close relationship between photosynthetic acclimation to low temperature and a capacity of antioxidant system in *L. multiflorum/F. arundinacea* introgresssion forms. The observed alterations in the activity of photosynthetic apparatus, including photoactivity and the Calvin cycle efficiency, under CA in the LFT form, were supported by a lower capacity of enzymatic antioxidant system and consequently, a higher content of ROS. This phenomenon, in turn, resulted in a lower stability of cellular membranes under stress conditions, characterized by higher T_EL50_ and lipid peroxidation in the LFT form. It cannot be excluded that in the efficient CA of photosynthetic process to avoid photoinhibition in the HFT introgression form, at least partially, higher abundance of Cor14b protein, was involved. Further research would be required to decipher the roles of cellular mechanisms described here, in the conditions of oscillating winter temperatures, including the possible sequence of cold acclimation, de-acclimation, and further cold re-acclimation processes. The hypothetical model demonstrating a part of cellular reactions to low temperature in the analyzed introgression forms is shown in Figure 10.

## Figures and Tables

**Table 1:** Sequences of primers and TaqMan probes for the target and reference genes in *Lolium multiflorum/Festuca arundinacea* introgression forms: plastid fructose-1,6-bisphosphate aldolase (pFBA), plastid phosphoglycerate kinase (pPGK), and actin, used in RT-qPCR analysis.

Gene	Primer Forward/Reverse	TaqMan Probe
*Actin*	GTCGAGGGCAACATATGCAA CCAGTGCTGAGCGGGAAT	TTCTCCTTGATGTCACGGAC
*pFBA*	GAGACGTTCTACTACATG GAGGAGCTTGAGAGTGTA	TGTTCCTGTCCTTGCACTCGG
*pPGK*	CCTTGGTTGAGGAAGATAA CAGCAATGACAACATCAG	CTGGCAACAACTCTCCTGGC

**Table 2:** Sequences of primers and TaqMan probes for the target and reference genes in *Lolium multiflorum/Festuca arundinacea* introgression forms: chloroplastic L-ascorbate peroxidase (APX), catalase (CAT), chloroplastic iron superoxide dismutase (Fe-SOD), mitochondrial manganese superoxide dismutase (Mn-SOD), chloroplastic copper/zinc superoxide dismutase (Cu/Zn-SOD), chloroplastic glutathione reductase (GR), chloroplastic glutathione peroxidase (GPX), and reference actin, used in RT-qPCR analyses.

Gene	Primer Forward/Reverse	TaqMan Probe
*Actin*	GTCGAGGGCAACATATGCAA CCAGTGCTGAGCGGGAAT	TTCTCCTTGATGTCACGGAC
*GR*	GGGGAGTACGACTACGACCT TCGTAAGTCCACCCAAAGCC	GGCGGCGTCAGGGCCTCGCGCTT
*GPX*	TCACTCGGCGGCCTGGAGAA TTCACAGTGCGGGCTTACGA	CTACGCCACCGCCGCCACGGAGAA
*APX*	CTCGTATCGCAGGAGCTCG TTGGGCCACTCGCTAATGTT	CGGCTGCGGCTGGAGATGCGACGGC
*Fe-SOD*	TCTATCTCGGCGGTTCTCCA CCGTTGTTGTAGGCCTCCTT	GCTCGACACCAGCCCCTTCTACGGCCA
*Cu/Zn-SOD*	CCAGAGCATCCTCTTCGCC ATTGATGGAGGTGGAAGCCG	TCGCTCCGCCTCGTCTCCGCCCCC
*Mn-SOD*	TTGACGCCGCTGTCTCTAAGG TTTATCCAACGCCAGCCACA	GCTTCCGCCGTCGTCCAACTCCAGGGC
*CAT*	GTTCACCTTCCTCTTCGACG AAGTCGAACCTGTCCTCGTG	ACTACCGCCACATGGATGGCTCCG

## Abbreviations

A	Net photosynthesis (assimilation of CO_2_)
APX	L-ascorbate peroxidase
CA	Cold acclimation
CAT	Catalase
CBF	C-repeat binding factor
Cor	Cold-regulated
Cu/Zn-SOD	Copper/zinc superoxide dismutase
E	Transpiration
EL	Electrolyte leakage
EDTA	Ethylenediaminetetraacetic acid
Fe-SOD	Iron superoxide dismutase
Fv/Fm	Maximum quantum yield of primary PSII photochemistry
FW	Fresh weight
GPX	Glutathione peroxidase
GR	Glutathione reductase
gs	Stomatal conductance
GSH	Reduced glutathione
HFT	High freezing tolerant
LFT	Low freezing tolerant
Mn-SOD	Manganese superoxide dismutase
NBT	Nitrobluetetrazolium
pFBA	Plastid fructose-1,6-bisphosphate aldolase
POD	Guaiacol peroxidase
PPFD	Photosynthetic photon flux density
pPGK	Plastid phosphoglycerate kinase
PS	Photosystem
ROS	Reactive oxygen species
RT-qPCR	Reverse transcription quantitative polymerase chain reaction
SDS	Sodium dodecyl sulfate
SOD	Superoxide dismutase
TBA	Thiobarbituric acid
TBARS	Thiobarbituric acid reactive substances
TCA	Trichloroacetic acid
T_EL50_	Temperature causing a 50% electrolyte leakage
WUE	Water use efficiency
